# Knowledge and willingness to donate kidney for transplantation among general population in Saudi Arabia

**DOI:** 10.1186/s12889-024-19766-2

**Published:** 2024-08-22

**Authors:** Mohammed Alshehri, Ibrahim Tawhari, Thekra S. Alqahtani, Alhanouf Y. Alqahtani, Marwah S. Al Jallal, Ghufran B. Asiri, Maymunah A. Alshahrani, Maryam A. Majrashi, Ahmed A. Khuzayyim, Fai D. Albishri, Wajan A. Alshahrani

**Affiliations:** 1 Nephrology Section, Internal Medicine Department, College of Medicine, King Khalid University, Abha, 61421 Saudi Arabia; 2 Medical Intern at College of Medicine, King Khalid University, Abha, 61421 Saudi Arabia

**Keywords:** Organ transplantation, Saudi Arabia, Public awareness, Transplantation ethics, Donor education

## Abstract

**Introduction:**

Kidney transplantation is the preferred treatment for end-stage renal disease (ESRD), offering a superior quality of life and extended survival compared to other renal replacement therapies. As the number of ESRD patients grows, so does the demand for organ transplants. The prevalence of ESRD is anticipated to escalate further due to the rising rates of diabetes mellitus (DM), hypertension (HTN), and obesity. Organ donation, particularly from living donors, remains the main source of transplants in the region, despite the notable underutilization of potential deceased donors’ organs. The objective of this research is to assess the level of knowledge, attitudes, and willingness to donate kidneys among the general population, a pivotal step in addressing the organ shortage crisis.

**Methods:**

This cross-sectional study was conducted in the Aseer region of Saudi Arabia using a previously validated questionnaire. The questionnaire collected demographic data and insights into general attitudes, knowledge, and beliefs about organ donation. Logistic regression was used to identify predictors of knowledge and willingness to donate.

**Results:**

The study involved 705 participants, predominantly young adults with a high level of education. Awareness of kidney donation was high, and knowledge about donation was broad, especially regarding religious permissibility and awareness of the donor registry. However, only 25% expressed willingness to donate their kidneys, and a 4% were already registered as donors. Furthermore, higher educational level was not associated with higher odds of knowledge or willingness to donate.

**Conclusion:**

Despite the considerable awareness, actual donor registration rates were low, highlighting the necessity for targeted educational interventions and a deeper understanding of the cultural and socioeconomic barriers that exist.

**Supplementary Information:**

The online version contains supplementary material available at 10.1186/s12889-024-19766-2.

## Introduction

Kidney transplantation has become a routine and the preferred treatment for End stage renal disease (ESRD), as it provides better quality of life and survival compared to renal replacement therapy [1, 2]. As per 2019 statistics, Saudi Arabia has the total number of 28,256 ESRD patients. Of these patients, 69% (19,522) were receiving hemodialysis (HD), while a quarter (7,188) had undergone transplantation, and a smaller fraction, 7% (1,546), were undergoing peritoneal dialysis (PD) [3]. The demand for organ transplants is growing due to an increase in non-communicable diseases, particularly diabetes and hypertension: the two leading causes of ESRD [4].

The first organ transplantation occurred in Saudi Arabia in 1979 with a living kidney transplantation. Since then, unlike several other countries, living-donors organs have remained the primary source for transplantation [5, 6] with no significant increase in the utilization of deceased donors’ organs. A national program for deceased donor transplantation is currently being developed at the national level to address the shortage of organs for transplantation. In 2022, a total of 149 intensive care centers across the country have contributed by reporting potential cases of deceased donors. In the Southern region, where the study population resides, 44 cases of potential deceased donors were reported, but only 6% (3 cases) were utilized. According to the Saudi Center of Organ Transplantation (SCOT), between 1986 and 2022, 67% of families approached for consent regarding potential transplantation from eligible brain-dead patients refused to consent for transplantation. This data is sourced from the Annual Report for Organ Transplantation in the Kingdom of Saudi Arabia (http://www.scot.gov.sa, accessed Dec 2023).

Understanding the reasons behind refusal is a complex task due to the multitude of contributing factors. The decision to donate a kidney is influenced by cultural beliefs, religious considerations, socio-economic status, and individual health perceptions [7]. Assessing the knowledge base of the population is crucial, as misconceptions and lack of awareness may contribute to apprehensions surrounding organ donation [8–10]. Educational interventions can be tailored to address specific gaps in understanding and promote a more informed and positive attitude toward kidney donation [11, 12]. Therefore, comprehending the level of knowledge and willingness within the general population to donate kidneys for transplantation is critical in addressing the organ shortage and enhancing outcomes for those with ESRD.

Our aim in this study is to evaluate knowledge and willingness to donate kidneys for transplantation among the general population in the region. We explored the factors that impact knowledge and willingness, including demographic and cultural factors, misconceptions about kidney donation, social support, and religious concerns.

## Methods

This study was a cross-sectional study conducted in the Aseer region of Saudi Arabia between July 1, December 31, 2023. The Aseer region is one of Saudi Arabia’s administrative regions, located in the southwestern part of the country. Its capital is Abha. This region is characterized by its diverse geography, which includes mountainous areas, coastal plains, and a mix of urban and rural populations. Aseer is culturally rich, with a population known for its traditional architecture, art, and festivals. We employed a questionnaire that has been previously validated in other publications and on a population similar to our target population [13]. We piloted the questionnaire with a sample of 15 participants to evaluate the clarity of the questions, the time required to complete the questionnaire and the response rate. The pilot study revealed the need for some linguistic modifications to better align with our study population. It also indicated that the average time to complete the questionnaire was 12–15 min and that the response rate was 25%. Using G*Power, we determined that the minimum required sample size was 279 participants, based on the following assumptions: a power of 95%, an alpha error of 5%, a two-tailed test, and an effect size of 0.1. This calculation was predicated on the assumption that 27.1% [14] of the population living in the Aseer region are willing to donate. To enhance the reliability and generalizability of the study, we doubled the initial sample size to 558 to account for stratification. Furthermore, we increased this number by 25% to accommodate a potential non-response rate, resulting in a final target sample size of approximately 698 participants. The inclusion criteria for this study were being aged 14 years or older, able to read and understand Arabic, and willing to provide informed consent electronically. Exclusion criteria included individuals who self-reported a diagnosis of ESRD. Additionally, incomplete survey responses were excluded from the final analysis to ensure data quality. The questionnaire was distributed online through various social media platforms, including Facebook, Twitter, and Instagram, to reach a broad and diverse segment of the general population in the Aseer region. To obtain consent, participants were provided with a detailed information sheet at the beginning of the questionnaire, outlining the study’s purpose, procedures, risks, and benefits. Participants were required to provide electronic consent by indicating their agreement to participate before they could proceed with the questionnaire. The questionnaire was distributed online to the general population through social media platforms. The questionnaire consists of two parts: the first includes basic demographic information regarding age, sex, education level, and being resident of Aseer region or not. The second part has several questions concerning: general inquiries about organ donation, the participant’s knowledge, attitude, beliefs, and intention to be an organ donor. This study’s protocol was approved by the Institutional Review Board at King Khalid University in Abha, Saudi Arabia (ECM#2023–2204).

The analysis of this cross-sectional study was done by the SPSS version 24, which is a software package from IBM Corp located in Armonk, New York, United States. All the variables were either categorical or dichotomous in nature therefore frequencies and percentages were used as estimates in our study. Furthermore, we identified the predictors of knowledge of organ donation, already registered as organ donor or ever donated any organ, blood or tissue, through the multivariable logistic regression which reported by the odds ratio (OR) and the 95% confidence interval (95%CI).

## Results

Our study sample size was 705 participants (Table 1). Most of our participants fall in the age of 18–34 years old (60.3%). Female prevalence was higher than males in our study (65.2% vs. 34.8%). Almost all of our participants had tertiary, university, or higher education (95.2%). Married participants’ prevalence was 51.5%, while 45.1% were not married and the rest were divorced or widowed (3.4%). Most participants were Saudi Arabian and are from Aseer region. Nearly a quarter of participants or a member of their family had a kidney disease.

**Table 1:** Characteristics of participants (*n* = 705)

Age	14–18	16 (2.3)
	18–24	248 (35.2)
	25–34	177 (25.1)
	35–44	130 (18.4)
	45–54	103 (14.6)
	55–64	30 (4.3)
	> 65	1 (0.1)
Sex	Female	460 (65.2)
	Male	245 (34.8)
Education	No education	3 (0.4)
	Primary	5 (0.7)
	Secondary	29 (4.1)
	Tertiary	155 (22.0)
	University degree or higher	513 (72.8)
Marital status	Married	363 (51.5)
	Not married	318 (45.1)
	Widow	17 (2.4)
	Divorced	7 (1.0)
Nationality	Non-Saudi	10 (1.4)
	Saudi	695 (98.6)
Region	Aseer	601 (85.2)
	Not Aseer	104 (14.8)
Do you or any family member had kidney disease	No	547 (77.6)
	Yes	158 (22.4)

Table 2 summarizes the answers to general inquiries about organ donation. Our survey of 705 individuals revealed that 92.9% were aware of renal donation, but only 12.5% had attended organ donation campaigns. While 15.7% were registered organ donors, 20.7% had previously donated organs or blood tissues, predominantly blood (95.4% of donors). Additionally, 22.4% reported personal or family history of kidney disease. Three out of the 705 participants have already donated kidneys.

**Table 2:** General inquiries about organ donation (*n* = 705)

Have you ever heard of the term kidney Donation	No	50 (7.1)
	Yes	655 (92.9)
Have you ever attended organ donation promotion campaigns in Aseer	No	566 (87.5)
	Yes	81 (12.5)
Are you registered as an organ donor	No	591 (84.3)
	Yes	111 (15.7)
Have you ever donated any organ or blood tissues	No	559 (79.3)
	Yes	146 (20.7)
What is it	Blood	124 (95.4)
	Plasma	1 (0.8)
	Platelet	1 (0.8)
	Stem cells	1 (0.8)
	Kidney	3 (2.3)
Do you or any family member had kidney disease	No	547 (77.6)
	Yes	158 (22.4)

### Knowledge about kidney donation

Of the respondents, 72.8% had a comprehensive understanding of organ donation, with 84% aware of Saudi Arabia’s donor registry. Islam’s permission for renal donation was acknowledged by 72.9%. Awareness of kidney donation safety stood at 46.6%, and 60.0% correctly identified 18 as the eligible age for kidney donation. Knowledge of the possibility of living kidney donation was high at 87.5%. In terms of personal connections, 36.0% knew a friend and 15.6% a family member who had donated a kidney.

### General attitude toward kidney donation

A substantial portion (86%) agreed that kidney donation is a positive act deserving promotion. Attitudes toward automatic inclusion in the organ donor registry revealed mixed views, with 49.7% agreeing and 25% disagreeing. Additionally, 69.2% strongly agreed that registering as a kidney donor could save a life. Regarding the influences on organ donation decision, 70.4% either strongly agreed or agreed that more information about kidney transplants would positively influence their decision. Similarly, 66.5% of respondents who strongly agreed or agreed that they would be more willing to register if they know their family wouldn’t object to organ donation. Additionally, understanding the registration process was crucial, as indicated by 65.5% of those who strongly agreed or agreed that knowing where to register would increase their willingness to become donors. Regarding religious perspective 74.6% of individuals either strongly agreeing or agreeing that more information about their religion’s viewpoint on kidney donation would impact their decision to register as donors. Table 3 summarizes the responses related to knowledge and attitudes of the study participants towards organ donation.

**Table 3:** Knowledge and attitude toward kidney donation (*n* = 705)

**Knowledge about kidney donation**		
What does organ donation mean to you	Transfer of tissues /blood/organs from a living donor to a patient in need	142 (21.8)
	Transfer of tissues or organ from a dead body to a patient in need	35 (5.4)
	All of the above	473 (72.8)
There is a donor registry in Saudi Arabia where people register during their life to donate organs after death. Have you heard about it	No	104 (14.8)
	Yes	399 (56.6)
	Partially	202 (28.7)
Does Islam allow organ donation especially kidney donation	No	164 (23.3)
	Yes	514 (72.9)
	Do not know	27 (3.8)
Do you know that donating a kidney is safe	No	98 (13.9)
	Yes	329 (46.6)
	May be	267 (37.8)
	Do not know	12 (1.7)
At what age can an individual register for Kidney donation	Any age	73 (10.4)
	18 above	423 (60.0)
	I do not know	209 (29.6)
Do you know that you can donate one of your two kidneys during your life to another person	No	88 (12.5)
	Yes	617 (87.5)
Do you know anyone who has donated kidney	Family member	110 (15.6)
	Friend	254 (36.0)
	No one	339 (48.1)
	Organ donor	1 (0.1)
	Celebrity	1 (0.1)
**Attitudes**		
Kidney donation is a good thing and should be promoted	Strongly agree	338 (47.9)
	Agree	269 (38.2)
	Neither agree nor disagree	93 (13.2)
	Disagree	4 (0.6)
	Strongly Disagree	1 (0.1)
Saudi Arabian as well as Non-Saudi Arabian residents should be automatically included on the Organ Donor register of Saudi Arabia with the ability to refuse if they wish	Strongly agree	181 (25.7)
	Agree	173 (24.5)
	Neither agree nor disagree	171 (24.3)
	Disagree	130 (18.4)
	Strongly Disagree	50 (7.1)
Registering as kidney donor could save somebody’s life	Strongly agree	488 (69.2)
	Agree	184 (26.1)
	Neither agree nor disagree	29 (4.1)
	Disagree	3 (0.4)
	Strongly Disagree	1 (0.1)
**I would be more willing to register as an organ donor**:		
If I knew more about what kidney transplant is and how it is done	Strongly agree	241 (34.2)
	Agree	255 (36.2)
	Neither agree nor disagree	164 (23.3)
	Disagree	32 (4.5)
	Strongly Disagree	13 (1.8)
If I knew that my family would have no objection to allowing donation of my organs at the time of my death	Strongly agree	258 (36.6)
	Agree	211 (29.9)
	Neither agree nor disagree	146 (20.7)
	Disagree	72 (10.2)
	Strongly Disagree	18 (2.6)
If I knew where I could register	Strongly agree	211 (29.9)
	Agree	251 (35.6)
	Neither agree nor disagree	185 (26.2)
	Disagree	47 (6.7)
	Strongly Disagree	11 (1.6)
If more information was available about the viewpoint of my religion with regard to kidney donation	Strongly agree	283 (40.1)
	Agree	243 (34.5)
	Neither agree nor disagree	143 (20.3)
	Disagree	26 (3.7)
	Strongly Disagree	10 (1.4)

### Beliefs

As shown in Table 4, 84.2% of the participants agreed that their donation will impact their afterlife positively, with 2.2% disagreeing. Regarding the concern of receiving adequate emergency care as registered donors, 29.3% agreed, while 29.0% disagreed.

86.1% agreed that kidney donation is a God-rewarded act, with 1.7% disagreeing. For increased donations with social support for the donor’s family, 51.2% agreed, and 8.8% disagreed.

Regarding the perception of kidney donor registration as time-consuming, 28.7% agreed, and 25.1% disagreed. Concerning the availability of registration opportunities, 29.8% agreed it was challenging, whereas 33.0% disagreed.

40.9% agreed there are concerns about unanswered questions during registration, and 20.3% disagreed. Concerns about the retrieval process causing disfigurement were agreed upon by 19.7%, with 38.4% disagreeing. Finally, 31.1% agreed that the organ procurement procedure is discouraging, while 24.0% disagreed.

### Influencing factors on knowledge or willingness to donate

University or higher education was associated with increased odds of being registered as a kidney donor. However, this was not statistically significant compared to the group with lower educational levels (OR: 1.46, 95% CI: 0.82–2.60) as shown in Table 5. People with higher education were more likely to have a reported history of donation, most commonly blood. Female respondents were less likely than male participants to have a previous history of organ, blood, or tissue donation (*p* < 0.001). Age, nationality, region, and a history of kidney disease were not associated with a greater likelihood of being registered for kidney donation or having a history of donation. However, married participants showed significantly lower odds of being registered as organ donors (OR: 0.42, 95% CI: 0.22–0.80, *p* = 0.008). Tables 1 and 5 supplementary.

**Table 4:** Beliefs

**Behavioral Beliefs**			
I think my donation whether living or after death is going to impact my life after death in a good way	Strongly agree	373 (52.9)	
	Agree	221 (31.3)	
	Neither agree nor disagree	95 (13.5)	
	Disagree	13 (1.8)	
	Strongly Disagree	3 (0.4)	
In case of an emergency doctors will not provide enough care if the patient is a registered kidney donor	Strongly agree	87 (12.3)	
	Agree	120 (17.0)	
	Neither agree nor disagree	294 (41.7)	
	Disagree	114 (16.2)	
	Strongly Disagree	90 (12.8)	
Kidney donation is an act which will be rewarded by God	Strongly agree	402 (57.0)	
	Agree	205 (29.1)	
	Neither agree nor disagree	86 (12.2)	
	Disagree	8 (1.1)	
	Strongly Disagree	4 (0.6)	
Kidney donation will increase if social support is provided to family of the deceased regardless of whether they donate or not	Strongly agree	142 (20.1)	
	Agree	219 (31.1)	
	Neither agree nor disagree	282 (40.0)	
	Disagree	42 (6.0)	
	Strongly Disagree	20 (2.8)	
**Normative Beliefs/ Subjective Norms**			
Whose opinion has a strong influence on your decisions	Family member	477 (67.7)	
	Religious leader	49 (7.0)	
	Friend	10 (1.4)	
	No one	136 (19.3)	
	Community	33 (4.7)	
**Control Beliefs/ Perceived Behavioral Control**			
Kidney donor registration is time consuming process	Strongly agree	50 (7.1)	
	Agree	152 (21.6)	
	Neither agree nor disagree	326 (46.2)	
	Disagree	122 (17.3)	
	Strongly Disagree	55 (7.8)	
You don’t find many opportunities to register as kidney donor in Saudi Arabia	Strongly agree	71 (10.1)	
	Agree	139 (19.7)	
	Neither agree nor disagree	262 (37.2)	
	Disagree	163 (23.1)	
	Strongly Disagree	70 (9.9)	
While registering for kidney donation you may not get answer for all your questions	Strongly agree	74 (10.5)	
	Agree	214 (30.4)	
	Neither agree nor disagree	274 (38.9)	
	Disagree	107 (15.2)	
	Strongly Disagree	36 (5.1)	
You are not healthy to donate	Strongly agree	61 (8.7)	
	Agree	164 (23.3)	
	Neither agree nor disagree	268 (38.0)	
	Disagree	152 (21.6)	
	Strongly Disagree	60 (8.5)	
Your age is not fit for donating your kidney	Strongly agree	46 (6.5)	
	Agree	114 (16.2)	
	Neither agree nor disagree	248 (35.2)	
	Disagree	211 (29.9)	
	Strongly Disagree	86 (12.2)	
Kidney retrieval process after death may cause body disfigurement	Strongly agree	48 (6.8)	
	Agree	91 (12.9)	
	Neither agree nor disagree	295 (41.8)	
	Disagree	160 (22.7)	
	Strongly Disagree	111 (15.7)	
Operation procedure for procuring organs is discouraging	Strongly agree	62 (8.8)	
	Agree	157 (22.3)	
	Neither agree nor disagree	317 (45.0)	
	Disagree	121 (17.2)	
	Strongly Disagree	48 (6.8)	
**Live Donation**			
You are worried that kidney donation might leave you weak and disabled	Strongly agree	153 (21.7)	
	Agree	240 (34.0)	
	Neither agree nor disagree	178 (25.2)	
	Disagree	94 (13.3)	
	Strongly Disagree	40 (5.7)	
I don t trust the health care system in Saudi Arabia and it is better to go abroad for kidney donation and transplantation	Strongly agree	37 (5.2)	
	Agree	73 (10.4)	
	Neither agree nor disagree	185 (26.2)	
	Disagree	180 (25.5)	
	Strongly Disagree	230 (32.6)	
**Donation after Death**			
Emotions of your family members while organ are being taken make you feel concerned	Strongly agree	229 (32.5)	
	Agree	209 (29.6)	
	Neither agree nor disagree	175 (24.8)	
	Disagree	53 (7.5)	
	Strongly Disagree	39 (5.5)	
**Intentions**			
Are you willing to register as a Kidney donor in Saudi Arabia	No	121 (17.2)	
	Yes	178 (25.2)	
	Did not decide	373 (52.9)	
	Already registered	33 (4.7)	

**Table 5:** Predictors of already registered as an organ donor and ever donated any organ, blood or tissue (*n* = 705)

Variables		Already registered as an organ donor				Ever donated any organ, blood, or tissue			
		OR	95% CI		P value	OR	95% CI		P value
			Lower	Upper			Lower	Upper	
Sex	Female	**Reference**							
	Male	0.708	0.438	1.145	0.159	9.620	6.094	15.186	**< 0.001**
Age	< 18	**Reference**							
	18–24	1.368	0.277	6.764	0.700	0.926	0.171	5.006	0.929
	25–34	2.095	0.411	10.674	0.373	1.317	0.235	7.391	0.754
	35–44	1.542	0.276	8.604	0.621	1.503	0.246	9.172	0.659
	45–54	0.844	0.130	5.484	0.859	1.352	0.213	8.590	0.749
	55–64	1.049	0.113	9.751	0.967	3.260	0.466	22.827	0.234
Education	Below university degree	**Reference**							
	University degree or higher	1.458	0.816	2.606	0.203	1.764	1.032	3.016	**0.038**
Nationality	Non-Saudi	**Reference**							
	Saudi	0.505	0.095	2.690	0.423	0.295	0.064	1.365	0.118
Marital status	Not married	**Reference**							
	Married	0.418	0.219	0.798	**0.008**	0.743	0.372	1.484	0.400
	Divorced	0.283	0.034	2.368	0.244	0.483	0.085	2.753	0.413
Region	Not Aseer	**Reference**							
	Aseer	0.879	0.474	1.630	0.682	1.192	0.638	2.226	0.582
Do you or any family member had kidney disease?	No	**Reference**							
	Yes	1.556	0.948	2.552	0.080	1.512	0.901	2.537	0.118

## Discussion

The present study aims to understand the landscape of kidney donation awareness, attitudes, and willingness among residents in the Aseer region of Saudi Arabia. Our study found that most participants were young adults, predominantly female, and highly educated, with most residing in the Aseer region of Saudi Arabia. Awareness of renal donation was high, yet participation in organ donation campaigns was low. A substantial portion of the respondents had a comprehensive understanding of organ donation and were aware of the national donor registry, with many acknowledging the permissibility of renal donation in Islam. Positive attitudes towards kidney donation were prevalent, with many recognizing its potential to save lives and viewing it as a commendable act. Concerns about registration processes, emergency care, and procedural barriers were noted. The study also revealed that gender and marital status significantly influenced donation history and willingness, indicating the need for targeted, sensitive approaches to encourage broader participation in organ donation.

### Sociodemographic of the participants and their association with knowledge

The demographic distribution of respondents reveals a predominant female participation. Similar to findings in other studies from Saudi Arabia, women tend to participate more in cross-sectional studies on health issues [15]. Several considerations explain why our study had a higher proportion of female volunteers (65%) than male donors. Cultural and societal conventions may influence giving habits, with men being more likely to engage in public contribution activities. Furthermore, differences in awareness, education, and personal or religious convictions across genders may influence donation decisions. Social and familial factors may also play a role, as men may face different pressures or support networks regarding organ donation. Furthermore, discrepancies in healthcare access or impediments experienced by men and women may contribute to the observed disparity. Understanding these factors provides useful information for establishing focused initiatives to boost donation rates across all populations. Additionally, a significant representation of individuals aged 18–24 years offers valuable insights into the attitudes of younger demographics towards kidney donation. Moreover, most respondents possess a bachelor’s degree or higher, indicating a potentially higher level of education within the sample. This factor could influence their awareness and perceptions of organ donation [16, 17].

### Level of awareness among the study participants

The study reports a high level of awareness among respondents, with 92.9% indicating familiarity with the term kidney donation. The responses to the knowledge questions reveal that the majority of respondents were able to provide correct answers, including the awareness about the national registry and the religious permissibility for organ donation. Previous studies from Saudi Arabia have shown variable levels of knowledge and understanding regarding organ donation [8–10, 14, 18–21]. These variations in findings across studies may be attributed to differences in study populations and the methods used to evaluate overall knowledge, highlighting the need for standardized tools and consistent methodologies in future research.

### Studied population position toward kidney transplantation

Approximately one-third of our study population was willing to register or had already registered as a kidney donor in Saudi Arabia. This willingness to donate varies significantly amongst studies conducted in Saudi Arabia. An online survey of 1245 participants reported willingness towards organ donation among 19.6% of the respondents [7]. Their willingness strongly depended on the acceptability of organ donation among their family members, similar to the findings of our study. Conversely, a study by Alnasyan et al. found a much higher rate of willingness towards organ donation, approximately 77% [15].

This finding was seen more in participants belonging to an older age group, above 40 years of age, whereas in our study the majority of the respondents were much younger and mostly belonged to the age group of 18 to 24 years. Variations in willingness may also reflect changes in cultural, educational, or social circumstances between studies. To overcome registration difficulties, younger individuals may require more targeted education and family interaction. In contrast, older people may have more established viewpoints or increased exposure to organ donation difficulties, which could explain their higher readiness. These data imply that, while younger people are eager to donate organs, focused treatments addressing family acceptability and broader educational efforts could help to increase organ donation rates even further. Understanding these demographic and contextual disparities is critical for developing effective organ donation campaigns for diverse communities.

### Association between knowledge and willingness to donate

A study by Alessa et al. [10] reported that the majority of participants (63%) were unwilling to donate organs, a finding attributed to a lack of knowledge about organ donation within the study population. Another survey conducted among science students in Saudi Arabia found that only a minority (26%) were willing to donate organs, which was associated with their awareness [14]. In comparison, our study participants demonstrated a greater awareness of organ donation; however, their willingness to donate was similar to that observed in the aforementioned studies. Furthermore, two other studies from Saudi Arabia reported a high rate of willingness toward organ donation, ranging between 63 and 67% [19, 20]. However, the study by Altraif et al. [20] revealed significant knowledge gaps. The authors pinpointed prevalent misconceptions about organ donation, including the belief that Islam prohibits donation and concerns about bodily disfigurement post-donation. Such misconceptions were not present in our respondents who had much more awareness regarding organ donation. This suggests that while awareness is an important factor, it may not be the sole determinant of willingness to donate. Factors such as personal beliefs, cultural attitudes, and family influence might also play significant roles in shaping individuals’ decisions about organ donation, indicating that increasing awareness alone may not be sufficient to boost donation rates.

Possessing a university degree or higher was significantly associated with increased odds of having donated an organ, blood, or tissue, compared to the group with lower educational levels (*p* = 0.038, Table 5). However, possessing a university degree or higher was not associated with a higher likelihood of being registered as a kidney donor. This observation aligns with findings from some previous studies in Saudi Arabia [8, 22]. In the contrary, studies from other countries do report that a higher level of education is associated with better knowledge and willingness toward organ donation [23, 24]. This disparity implies that, while education may alter past donation behaviors, it may not always result in greater registration rates for organ donation. It emphasizes the necessity to investigate variables other than education that may influence registration and readiness to donate, such as cultural influences, personal values, or unique hurdles to registration.

### Implication of the study

Our findings have several important implications for increasing kidney donation rates in Saudi Arabia’s Aseer region. Despite increased awareness, active participation in organ donation campaigns remains low. Public health initiatives should focus on providing clear information about the donation process. Younger and more educated people are more willing to donate, whereas married people are less likely; specialized messaging addressing each group’s specific issues is required. Gender-sensitive strategies are also required, as female donors are less likely to provide. Given the importance put on religious beliefs, contacting religious leaders and employing culturally sensitive messages can promote donations. Simplifying the registration procedure and providing clear, thorough information can help to lower procedural obstacles. It is critical to establish trust in the healthcare system by addressing concerns regarding emergency care and retrieval procedures. Highlighting social support and acknowledging donor families can help to increase donations. To boost donation rates and public health results, policymakers could speed registration, provide donor support, and communicate culturally and religiously relevant public health messaging.

### Strengths and limitations

The strength of this study lies in its sample size and use of an already validated questionnaire to gather data. However, the present study has a few limitations that warrant consideration. The cross-sectional design provides a snapshot of attitudes and knowledge at a specific point, limiting the ability to infer causation or changes over time [20]. The reliance on self-reported data may introduce the possibility of social desirability bias, where respondents may provide answers, they perceive as socially acceptable rather than reflecting their true attitudes [21]. Additionally, the study’s focus on the Aseer region may limit the generalizability of findings to the broader Saudi population, as regional variations in attitudes toward organ donation have been observed [5, 6]. Finally, there are restrictions to distributing the questionnaire via social media channels. These platforms naturally blind the researcher to respondents’ identities, making it impossible to confirm that participants meet all inclusion requirements and do not have ESRD. Furthermore, the voluntary nature of involvement may result in self-selection bias, in which people with specific interests or qualities are more likely to reply. Twitter, Facebook, and WhatsApp were selected for this study due to their high usage rates and ability to reach a large audience in the Aseer region. Nonetheless, the results of our study can be utilized effectively to design targeted educational programs to increase the rates of organ donation in the future.

## Conclusion

This study shows a high level of awareness and good understanding of kidney donation among the study’s participants. It, however, shows that people with higher education are not more than others in terms of willingness to donate or register as kidney donors.

### Electronic supplementary material

Below is the link to the electronic supplementary material.

Supplementary Material 1

## Data Availability

The datasets generated and/or analyzed during the current study are available from the corresponding author on reasonable request.

## Abbreviations

ESRD: End-Stage renal disease
HD: Hemodialysis
PD: Peritoneal dialysis
SCOT: Saudi center of organ transplantation
SPSS: Statistical package for the social sciences
OR: Odds ratio
CI: Confidence interval
