# Supplementary material for: Knowledge and willingness to donate kidney for transplantation among general population in Saudi Arabia
Source: BMC Public Health. 2024 Aug 22;24:2277. doi: 10.1186/s12889-024-19766-2 (PMC11340056; doi:10.1186/s12889-024-19766-2)
Supplement: Supplementary file 1 — Supplementary Material 1 [file 12889_2024_19766_MOESM1_ESM.docx]

| Table 1s : Unadjusted predictors of already registered as an organ donor and ever donated any organ, blood or tissue (n= 705) | | | | | | | | | |
| --- | --- | --- | --- | --- | --- | --- | --- | --- | --- |
| Variables | | Already registered as an organ donor | | | | Ever donated any organ, blood or tissue | | | |
|  |  | OR | 95% CI | | P value | OR | 95% CI | | P value |
|  |  |  | Lower | Upper |  |  | Lower | Upper |  |
| Sex | Female | **Reference** | | | | | | | |
|  | Male | 0.619 | 0.393 | 0.976 | **0.039** | 9.597 | 6.285 | 14.653 | **<0.001** |
| Age | <18 | **Reference** | | | | | | | |
|  | 18-24 | 1.637 | 0.360 | 7.448 | 0.524 | 1.552 | 0.341 | 7.069 | 0.570 |
|  | 25-34 | 2.044 | 0.446 | 9.372 | 0.358 | 1.604 | 0.348 | 7.402 | 0.545 |
|  | 35-44 | 0.845 | 0.174 | 4.110 | 0.835 | 1.750 | 0.374 | 8.185 | 0.477 |
|  | 45-54 | 0.433 | 0.079 | 2.360 | 0.333 | 2.364 | 0.503 | 11.101 | 0.276 |
|  | 55-64 | 0.500 | 0.064 | 3.932 | 0.510 | 6.125 | 1.181 | 31.768 | **0.031** |
| Education | Below university degree | **Reference** | | | | | | | |
|  | University degree or higher | 1.855 | 1.108 | 3.105 | **0.019** | 1.661 | 1.064 | 2.593 | **0.026** |
| Nationality | Non-Saudi | **Reference** | | | | | | | |
|  | Saudi | 0.744 | 0.156 | 3.551 | 0.711 | 0.385 | 0.107 | 1.383 | 0.144 |
| Marital status | Not married | **Reference** | | | | | | | |
|  | Married | 0.374 | 0.238 | 0.588 | **<0.01** | 1.225 | 0.848 | 1.771 | 0.279 |
|  | Divorced | 0.225 | 0.029 | 1.720 | 0.151 | 0.548 | 0.123 | 2.453 | 0.432 |
| Region | Not Aseer | **Reference** | | | | | | | |
|  | Aseer | 0.879 | 0.474 | 1.630 | 0.682 | 0.773 | 0.449 | 1.333 | 0.355 |
| Do you or any family member had kindey disease? | No | **Reference** | | | | | | | |
|  | Yes | 1.275 | 0.800 | 2.032 | 0.307 | 0.965 | 0.622 | 1.497 | 0.872 |
